# Supplementary figures and images for: The Finnic Peoples of Russia: Genetic Structure Inferred from Genome-Wide and Y-Chromosome Data
Source: Genes (Basel). 2024 Dec 17;15(12):1610. doi: 10.3390/genes15121610 (PMC11675159; doi:10.3390/genes15121610)

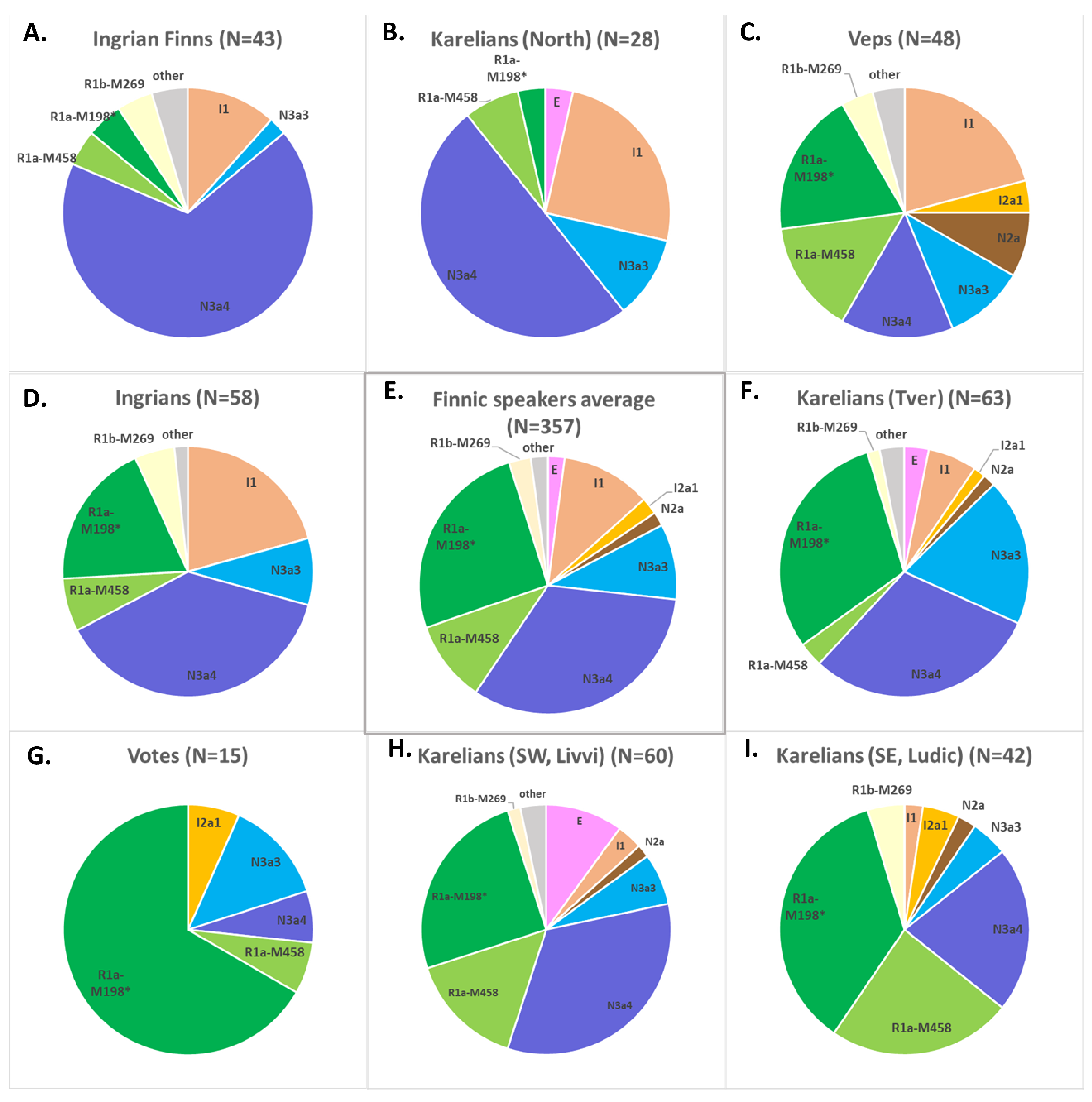

Supplement: Supplementary file 1 [file genes-15-01610-s001.zip › Figure S1.tif]

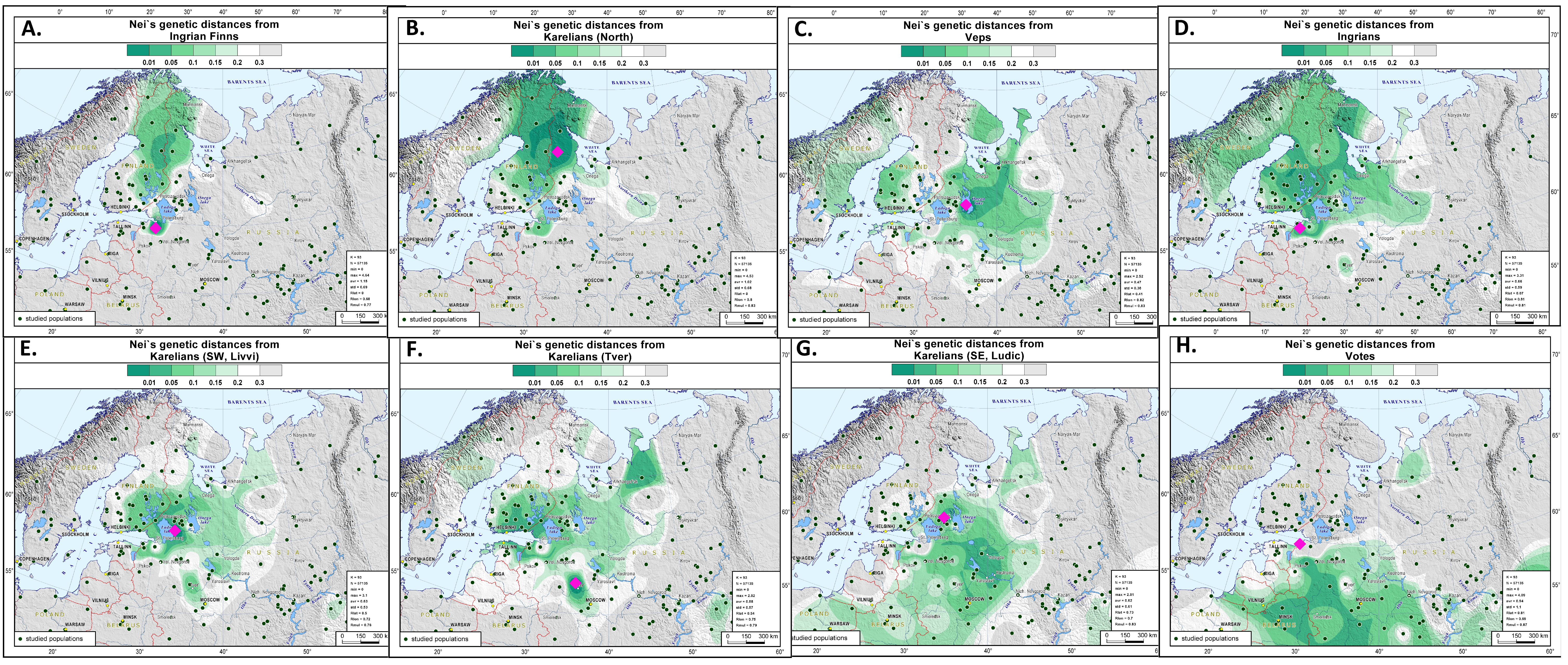

Supplement: Supplementary file 1 [file genes-15-01610-s001.zip › Figure S2.tif]

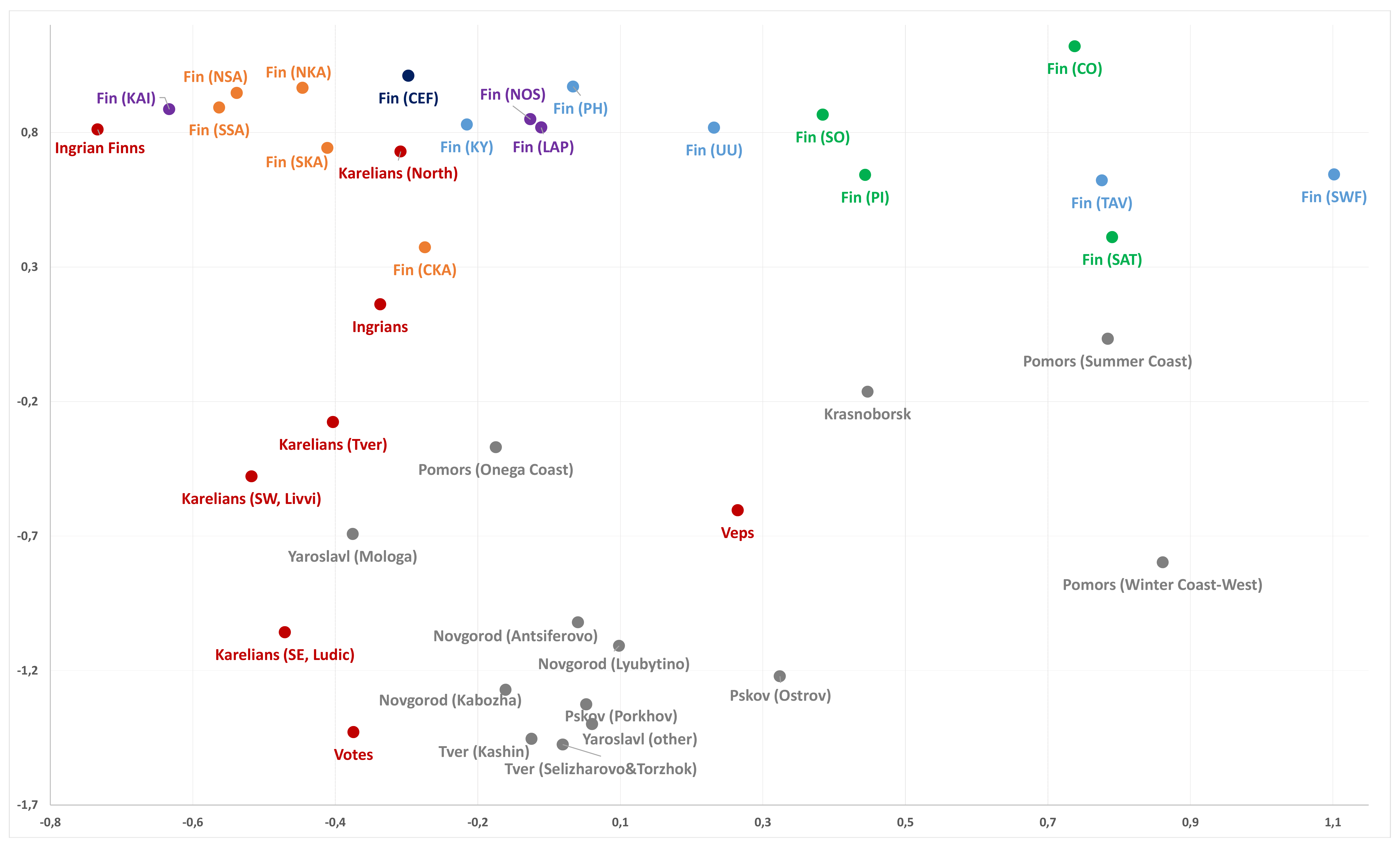

Supplement: Supplementary file 1 [file genes-15-01610-s001.zip › Figure S3.tif]
